# Loss in life expectancy after a colon cancer diagnosis by socioeconomic group: does the indicator of socioeconomic position matter?

**DOI:** 10.1186/s12889-025-25610-y

**Published:** 2025-11-28

**Authors:** Elisavet Syriopoulou, Alexander Miething, Erik Osterman, Caroline Nordenvall, Therese M-L Andersson

**Affiliations:** 1 Department of Medical Epidemiology and Biostatistics, Karolinska Institutet, PO Box 281, Stockholm, SE-17177 Sweden; 2 Department of Public Health Sciences, Stockholm University, Stockholm, Sweden; 3 Department of Surgery, Gävle Hospital, Gävle, Sweden; 4 Department of Molecular Medicine and Surgery, Karolinska Institutet, Stockholm, Sweden; 5 Department of Pelvic cancer, colorectal surgery unit, Karolinska University Hospital, Stockholm, Sweden

**Keywords:** Cancer disparities, Cancer survival, Loss in life expectancy, Socioeconomic position, Colon cancer

## Abstract

**Background:**

Loss in life expectancy (LLE) by socioeconomic position (SEP) is obtained to explore cancer disparities. Often education or income are used to determine SEP. However, the low income of some older individuals or females within households with greater overall resources may not accurately reflect their SEP, potentially leading to biased estimates. Our study investigates how various conceptualizations of SEP influence the extent of these disparities.

**Methods:**

Data included all colon cancer diagnoses in Sweden between 2008 and 2016. We estimated LLE using different SEP indicators: education, individual income, part of household income. Household income accounts for individuals with low individual income but within high-income households. We allocated patients to income groups creating quartiles, overall or separately by sex, age-groups, or both, to account for SEP misclassification. We analysed data using flexible parametric survival models.

**Results:**

Standardised LLE varied from 5.05 to 5.62 years for the lowest to the highest education, resulting in a 0.57-year difference. When income was used to determine SEP, LLE was similar with disparities ranging from 0.29 to 0.77 across definitions. Age and sex specific LLE disparities varied more across definitions. Disparities between 60-year-old females in the highest-lowest income groups were 0.94 when using household income and − 0.17 when using individual income.

**Conclusions:**

When studying LLE by SEP, thoughtful consideration must be given to selecting the most suitable indicator for conceptualizing SEP. The choice should align with the specific SEP pathway of interest and address potential misclassification concerns relevant to the study population.

**Supplementary Information:**

The online version contains supplementary material available at 10.1186/s12889-025-25610-y.

## Background

Several studies have explored disparities in prognosis after a cancer diagnosis by socioeconomic groups [1–3]. Socioeconomic position (SEP) reflects an individual’s position in society and has been shown to greatly impact health outcomes, including cancer prognosis. Socioeconomic disparities have been reported in various measures used to quantify cancer prognosis, such as relative survival [4, 5] and loss in life expectancy [6, 7]. Relative survival is the proportion who survived their cancer up to a specific time point e.g. 5-year relative survival [8]. Loss in life expectancy (LLE) is the reduction in life expectancy following the cancer diagnosis (average years lost) and captures the lifetime impact of cancer [9]. The reasons for the observed disparities are likely multifactorial and factors that have been suggested before include tumour stage, health-related behaviors, comorbidities and treatment modalities [10, 11]. Moreover, previous studies have shown that low participation in breast, cervical, and colorectal cancer screening is associated with lower SEP [12–15]. As screening attendance leads to diagnosis in earlier stages this could result in better cancer outcomes among those with a higher SEP for cancers in which screening is available.

The definition of SEP varies between studies and countries and potential limitations of the chosen definition are often overlooked in cancer studies. For instance, in England, the most widely used index is an area-level measure called The Index of Multiple Deprivation, which is based on seven domains of deprivation that are combined and weighted to produce an overall measure of multiple deprivation experienced by people living in an area at the time of their diagnosis [6, 16, 17]. In other countries, including Sweden, the highest education level achieved is often used as an indicator for SEP [7, 18, 19]. Another suggested indicator is income [1, 20, 21]. Different indicators capture different pathways that affect health outcomes, thus careful consideration of the measures used to determine SEP is required. The highest education level attained is often used as a proxy for the patients’ health literacy, ability to navigate the health system effectively and to process the information given, and has been found to be associated with health awareness [22]. Differences in cancer outcomes between education groups may, thus, reflect the varying levels of health literacy, access to healthcare, and preventive behaviors. Income may affect health outcomes through the ability to have a healthier lifestyle (e.g. good housing, safe neighbourhoods) and easy access to health resources. In addition to these material aspects, income can also be an indicator of an individual’s relative position in society as well as the degree of integration into society [23, 24]. Differences in cancer outcomes between income groups may, thus, reflect disparities in lifestyle, social support networks as well as access to health resources. Interestingly, disparities in health outcomes have been reported even in countries with universal healthcare access, like Sweden [21, 25, 26].

The following types of income can be used to allocate individuals into income groups: an individual’s disposable income, household income or part of household income that accounts for the number of individuals in a household [27]. Regardless of the measure used, there is a limitation as older retired individuals may be classified more often to a lower SEP group due to having a lower income compared to the working population although they may be part of a higher SEP group, and the same also applies to females [28]. On one hand, older individuals have lower survival and potential misclassification to lower SEP group may result in larger differences between the highest and lower income groups (so the true differences may be overestimated). On the other hand, females often have better survival than males, and misclassification to lower SEP group may result in smaller differences between the least and most affluent groups (the true difference may be underestimated). Allocating patients to income groups using quartiles’ cut-offs created separately by sex and age groups can possibly help address this limitation.

This study explored how different education- and income-based indicators and conceptualisations of SEP influence the estimates of relative survival or life expectancy measures, using data on colon cancer in Sweden. As females and older individuals are misclassified to a lower SEP under certain SEP definitions, we studied the impact of this misclassification on the estimates of interest. We also explored how the disparities between the highest and lowest SEP vary in magnitude under each SEP conceptualisation.

## Methods

### Data resources

Data originated from the Colorectal Cancer Database (CRCBaSe), a register-linkage of the Swedish Colorectal Cancer Registry and national registries at the National Board of Health and Welfare, Statistics Sweden and the Swedish Social Insurance Agency [29]. Data included all adults with a first-time diagnosis of colon cancer in Sweden between 2008 and 2016 and follow-up time to the end of 2017. Income and education were also available in the CRCBaSe through linkage with the Longitudinal Integrated Database for Health Insurance and Labour Market Studies [30], and were available between 2005 and 2016. Data includes comparators from the general population matched to the cancer population on birth year, sex, year of diagnosis, and county. There was information on the highest attained education, individual disposable income (IDI) and family disposable income per consumption unit (obtained as the sum of the disposable income of all family members divided with the consumption weight that applies to the household) [30]. We will refer to family disposable income per consumption unit as *part of household income* (PHI).

### Definition of socioeconomic position (SEP)

We created the exposure of interest as categories of the highest attained education or income groups. Specifically, we defined SEP in the 8 following ways: 1) highest attained education (completed: <9 years of compulsory education, 9 years of compulsory education, secondary education, tertiary education), 2) IDI groups using cut-offs based on quartiles using the whole population of controls (overall) 3) IDI groups using cut-offs based on quartiles created separately for patients below and above 65 years old at diagnosis, 4) IDI groups using cut-offs based on quartiles created separately within age-groups (5-year groups apart from the youngest (< 30 years) and the oldest (90 + years)), 5) IDI groups using cut-offs based on quartiles created separately by sex 6) IDI groups using cut-offs based on quartiles created separately by sex and the 65 years of age cut-off, 7) PHI groups using cut-offs based on quartiles created using everyone (overall) and (8) PHI groups using cut-offs based on quartiles created separately for patients above and below 65 years old. In definition (2), IDI quartiles were created based on everyone, and older individuals and females tended to be part of the lowest SEP. In definition (3), the older retired individuals were compared to similar individuals and more older individuals ended up on higher SEP. The cut-off points for all quartiles were based on the matched controls that are a proxy for the general population (Supplementary Material B). Hence, the income categories for the cancer population are not of equal size, which is in line with what is reported by several studies (incidence will be higher among certain SEP groups) [31, 32]. The highest attained education the year prior to the diagnosis year was chosen. If this was missing, the latest available information was used. For income, the average of the last 3 years prior to the diagnosis year was used. Income was adjusted for inflation [33].

### Relative survival and loss in life expectancy

Relative survival is the proportion of cancer patients that will still be alive at a specific time after their diagnosis in a hypothetical world where it is not possible to die from other causes [34, 35]. It is estimated by comparing the all-cause survival of a cancer population to the expected survival in the absence of cancer. The expected mortality rates of each patient are obtained by population lifetables which are stratified by sex, age, year. The cause of death is not utilised and issues with inaccurate causes of death are avoided.

Loss in life expectancy is the reduction in life expectancy following a cancer diagnosis and is defined as the difference between the life expectancy in the absence of colon cancer (based on expected mortality rates from population lifetables) and the life expectancy of the colon cancer population [9]. In this way, the impact of the cancer diagnosis on the whole remaining lifespan and in the presence of other competing cause of deaths is obtained, as opposed to looking only at a particular point in time as it is the case with relative survival [36].

### Constructing lifetables stratified by SEP

Having appropriate lifetables for the expected mortality rates is essential to get unbiased estimates. Since both cancer and other cause mortality vary by SEP it was important to use expected mortality rates stratified by year, sex, age and SEP for this analysis. Such lifetables were not available for Sweden and were constructed by further adjusting readily available expected mortality rates for Sweden stratified by sex, age and year from the Human Mortality Database [37] by SEP [38]. For this, data on the matched comparators including SEP information was used and 8 updated lifetables were constructed (one for each of the eight SEP definitions of interest).

### Statistical analysis

For each SEP definition, we fitted a flexible parametric survival model [9] that included sex, age (continuous, non-linear using splines, 3 degrees of freedom(df)) and SEP. The baseline excess hazard was modelled using 5 df. We allowed time-dependent effects for all variables. Interactions were also included between sex and SEP, and SEP and age at diagnosis to allow for a different effect of SEP in males and females as well as across age at diagnosis.

For each SEP group, we obtained age and sex standardised estimates of (i) 1, 5 and 10-year relative survival, (ii) life expectancy (LE) in the general population, (iii) life expectancy after a colon cancer diagnosis and (iv) loss in life expectancy (LLE) after the cancer diagnosis. We also obtained age- and sex-specific estimates of all the above measures. We calculated the disparities between the highest and lowest SEP as the difference in the estimates between the highest and lowest income groups (for income-based indicators), and the difference between tertiary and < 9 years of compulsory education (for education). All statistical analyses were performed using Stata 16 [39].

## Results

The analysis included 39,526 colon cancer patients, 19,903 (50.4%) of whom were females. The median age at diagnosis was 73 and 75 years for males and females, respectively. Supplementary Table S1 shows comparative tables for the number of patients in each SEP across different definitions.

### Age and sex standardised estimates

**Fig. 1:**
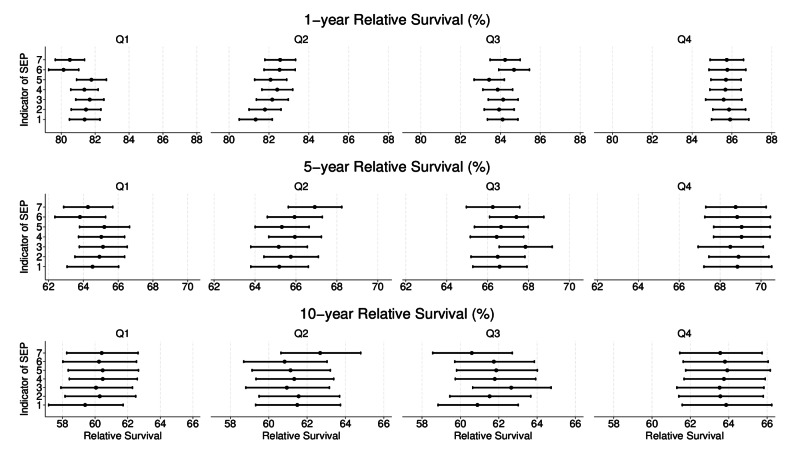
One, five and ten-year age and sex standardised relative survival (%), with 95% confidence intervals, by income group (Q1-Q4) across different ways used to create income quartiles as an indicator for socioeconomic position (SEP). Q4 refers to the highest and Q1 to the lowest income groups. Indicators 1 to 7 on the y-axis correspond to the following: 1=IDI (overall), 1=IDI (by age cut-off), 3=IDI (by sex), 4=IDI (by age cut-off & sex), 5=IDI (by age-groups), 6=PHI (overall), 7=PHI (by age cut-off), with IDI=individual disposable income, PHI=part of household income

When IDI was used as a proxy for SEP and the quartiles were created based on all patients, the absolute difference in age and sex standardised relative survival between the highest and lowest income groups was 5, 4 and 4% points at 1, 5 and 10 years, respectively (Fig. 1, Supplementary Table S2). In general, standardised estimates of relative survival were not overly sensitive to the way that the quartiles were created, and the largest change was 2 percentage points (Fig. 1, Supplementary Figure S1).

**Fig. 2:**
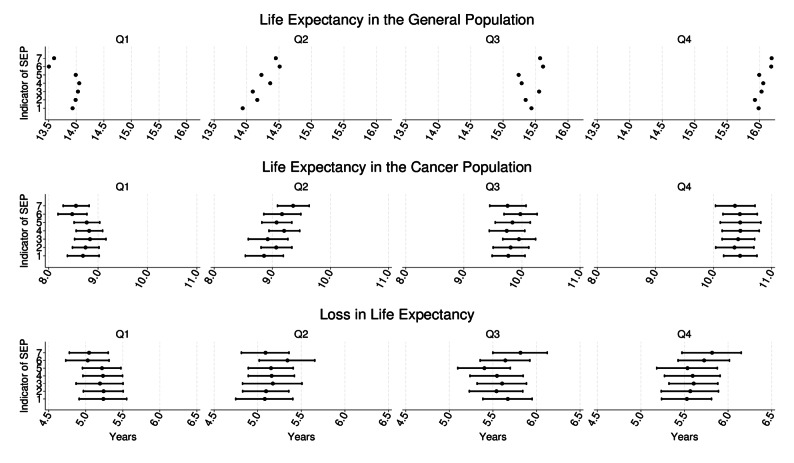
Age and sex standardised life expectancy with and without colon cancer (in years) and loss in life expectancy after a cancer diagnosis, with 95% confidence intervals, by income group (Q1-Q4) across different ways used to create income quartiles as an indicator for socioeconomic position (SEP). Q4 refers to the highest and Q1 to the lowest income groups. Indicators 1 to 7 on the y-axis correspond to the following: 1=IDI (overall), 1=IDI (by age cut-off), 3=IDI (by sex), 4=IDI (by age cut-off & sex), 5=IDI (by age-groups), 6=PHI (overall), 7=PHI (by age cut-off), with IDI=individual disposable income, PHI=part of household income

Age and sex standardised metrics of life expectancy showed small variation across different SEP definitions (Fig. 2, Supplementary Table S3). The life expectancy of the general population was 13.93 and 15.98 years on average for the lowest and highest income groups, i.e., 2.05 years difference when IDI was used (overall). This difference varied from 1.94 years to 2.67 across SEP definitions. Small variation was also observed for the difference in the life expectancy of the cancer population, with differences between SEP groups ranging from 1.59 to 1.98 years. For the LLE, the largest difference (between the highest and lowest income groups) was 0.77 years (when quartiles were created by sex using PHI) and the smallest 0.29 years (when using IDI).

### Age- and sex-specific estimates

Different ways used to create income quartiles resulted in larger variations for age-specific estimates of relative survival, especially for individuals diagnosed at a younger age. For instance, the absolute difference in 10-year relative survival between 60-years-old males in the highest and lowest income groups, was 5% points when income quartiles were based on PHI and created separately using age cut-off of 65 years (Table 1). However, when IDI was used (overall), there was a 9% points absolute difference in 10-year survival between the highest and lowest income groups. For individuals diagnosed at 75 years old, disparities between income groups were in general smaller (Supplementary Table S4).

**Table 1:** One, five and ten-year relative survival (%) for a patient diagnosed with colon cancer at 60 years old by income group (from the lowest (Q1) to the highest (Q4) income) and across different ways to create the income quartiles, by sex. The difference in relative survival between the highest (Q4) and lowest (Q1) income groups is also provided. 95% confidence intervals are given in the parentheses

Years After Diagnosis	Indicator for SEP	Q1	Q2	Q3	Q4	Difference Q4-Q1
Males						
1	IDI (overall)	82 (80–84)	83 (81–85)	87 (86–88)	88 (87–89)	6
1	IDI (by age cut-off)	83 (81–84)	85 (84–87)	87 (85–88)	88 (87–89)	6
1	IDI (by sex)	83 (81–84)	85 (84–87)	87 (86–88)	88 (87–89)	5
1	IDI (by age cut-off & sex)	83 (82–84)	86 (85–87)	87 (86–88)	89 (88–90)	6
1	IDI (by age-groups)	83 (81–84)	86 (85–87)	87 (86–88)	88 (87–89)	6
1	PHI (overall)	81 (80–83)	84 (83–86)	87 (86–88)	88 (87–89)	6
1	PHI (by age cut-off)	82 (81–84)	86 (84–87)	87 (86–88)	88 (87–89)	6
5	IDI (overall)	61 (57–64)	63 (59–66)	67 (64–69)	69 (67–71)	9
5	IDI (by age cut-off)	61 (58–64)	67 (64–69)	66 (64–69)	69 (67–71)	8
5	IDI (by sex)	61 (58–64)	65 (62–68)	68 (66–70)	68 (66–70)	7
5	IDI (by age cut-off & sex)	62 (59–64)	67 (65–69)	66 (64–69)	70 (68–72)	8
5	IDI (by age-groups)	61 (58–64)	67 (64–69)	68 (66–70)	69 (67–71)	8
5	PHI (overall)	60 (57–64)	64 (61–67)	66 (64–69)	69 (67–70)	8
5	PHI (by age cut-off)	61 (59–64)	67 (64–69)	66 (64–68)	68 (66–70)	7
10	IDI (overall)	53 (49–57)	57 (53–61)	59 (56–62)	62 (60–65)	9
10	IDI (by age cut-off)	54 (51–58)	61 (57–64)	59 (56–62)	62 (59–64)	8
10	IDI (by sex)	53 (50–57)	59 (55–63)	61 (58–63)	61 (59–64)	8
10	IDI (by age cut-off & sex)	54 (51–58)	60 (57–63)	59 (56–62)	63 (60–65)	8
10	IDI (by age-groups)	54 (50–57)	61 (58–64)	61 (59–64)	62 (59–65)	8
10	PHI (overall)	55 (51–59)	56 (53–60)	59 (56–61)	62 (59–64)	7
10	PHI (by age cut-off)	55 (52–59)	61 (57–64)	58 (55–61)	61 (58–64)	5
Females						
1	IDI (overall)	85 (83–86)	84 (82–85)	87 (86–88)	88 (87–90)	4
1	IDI (by age cut-off)	84 (83–85)	86 (85–88)	87 (86–88)	89 (88–90)	5
1	IDI (by sex)	85 (83–87)	85 (83–86)	86 (84–87)	88 (87–89)	3
1	IDI (by age cut-off & sex)	84 (83–85)	86 (85–88)	86 (85–87)	88 (87–89)	4
1	IDI (by age-groups)	84 (83–86)	87 (86–88)	88 (87–89)	89 (88–90)	5
1	PHI (overall)	83 (81–85)	85 (83–86)	87 (86–88)	88 (87–89)	5
1	PHI (by age cut-off)	84 (82–85)	86 (84–87)	87 (86–88)	88 (87–90)	5
5	IDI (overall)	66 (63–69)	65 (62–68)	68 (65–70)	70 (68–72)	4
5	IDI (by age cut-off)	64 (62–67)	69 (67–71)	68 (66–70)	70 (68–73)	6
5	IDI (by sex)	67 (64–70)	65 (62–68)	67 (64–69)	70 (68–71)	3
5	IDI (by age cut-off & sex)	65 (63–68)	69 (66–71)	66 (64–69)	70 (68–73)	5
5	IDI (by age-groups)	64 (62–67)	69 (67–71)	70 (68–72)	71 (68–73)	6
5	PHI (overall)	64 (61–67)	66 (63–69)	68 (66–70)	69 (67–71)	5
5	PHI (by age cut-off)	64 (62–67)	68 (66–70)	67 (65–69)	70 (68–72)	6
10	IDI (overall)	61 (58–65)	61 (58–65)	62 (59–65)	65 (63–68)	4
10	IDI (by age cut-off)	59 (56–62)	65 (62–68)	63 (60–66)	65 (62–68)	6
10	IDI (by sex)	62 (58–66)	61 (58–65)	61 (58–64)	65 (62–67)	3
10	IDI (by age cut-off & sex)	60 (57–63)	64 (61–67)	61 (58–65)	65 (62–68)	5
10	IDI (by age-groups)	59 (57–62)	64 (62–67)	65 (62–68)	66 (62–69)	6
10	PHI (overall)	60 (57–64)	61 (57–64)	63 (60–65)	64 (61–66)	4
10	PHI (by age cut-off)	60 (57–64)	63 (60–66)	61 (58–64)	65 (62–68)	4

*IDI* individual disposable income, *PHI* part of household income, *SEP* socioeconomic position

Age- and sex-specific measures of life expectancy varied more compared to the standardised ones. Consider as an example the estimates obtained when allocating individuals in income quartiles based on PHI (overall) (Table 2). A 60-year-old female has on average a life expectancy of 23.35 years and 27.23 years if she is in the lowest and highest income group, respectively. A colon cancer diagnosis will reduce the life expectancy of both females to 14.40 years and 17.34 years, respectively. Thus, a female in the lowest income group loses 8.95 years, while a female in the highest income loses 9.89 years (almost 1 year more). When repeating the analysis using IDI and creating income quartiles by age-groups, the disparity between the two females was reversed, with the 60-year-old from the lowest income group losing 0.46 years more than the female in the highest income group. Smaller variation was observed for older individuals (Table 3).

**Table 2:** Life expectancy with and without colon cancer (in years) for a 60-year-old individual and loss in life expectancy after the cancer diagnosis by income group (from the lowest (Q1) to the highest (Q4) income) and across different ways to create the income quartiles, by sex. The difference between the highest (Q4) and lowest (Q1) income groups is also provided. 95% confidence intervals are given in the parentheses

Indicator for SEP	Q1	Q2	Q3	Q4	Difference Q4-Q1
Males					
Life expectancy in the general population					
IDI (overall)	19.85	20.20	23.07	25.12	5.27
IDI (by age cut-off)	19.94	21.15	23.19	24.94	5.00
IDI (by sex)	19.80	21.35	24.05	25.23	5.42
IDI (by age cut-off & sex)	19.94	22.16	23.96	25.24	5.30
IDI (by age-groups)	19.83	21.53	23.31	25.07	5.24
PHI (overall)	19.51	21.35	23.56	25.12	5.60
PHI (by age cut-off)	19.81	21.58	23.59	25.09	5.28
Life expectancy in the cancer population					
IDI (overall)	10.93 (10.16–11.77)	11.84 (11.07–12.67)	13.72 (13.03–14.45)	15.60 (14.98–16.26)	4.67
IDI (by age cut-off)	11.18 (10.52–11.90)	13.13 (12.47–13.83)	13.98 (13.27–14.72)	15.39 (14.67–16.13)	4.20
IDI (by sex)	10.98 (10.28–11.74)	12.92 (12.14–13.75)	14.54 (13.86–15.25)	15.40 (14.72–16.12)	4.42
IDI (by age cut-off & sex)	11.25 (10.64–11.88)	13.55 (12.89–14.25)	14.35 (13.62–15.13)	15.74 (14.98–16.54)	4.49
IDI (by age-groups)	11.04 (10.40–11.72)	13.38 (12.74–14.05)	14.47 (13.79–15.19)	15.49 (14.74–16.28)	4.45
PHI (overall)	11.13 (10.42–11.89)	12.36 (11.58–13.18)	13.88 (13.19–14.61)	15.48 (14.82–16.17)	4.35
PHI (by age cut-off)	11.38 (10.74–12.06)	13.35 (12.68–14.06)	13.79 (13.08–14.55)	15.17 (14.44–15.94)	3.79
Loss in life expectancy					
IDI (overall)	8.92 (8.08–9.69)	8.36 (7.53–9.13)	9.35 (8.62–10.04)	9.52 (8.86–10.14)	0.60
IDI (by age cut-off)	8.76 (8.05–9.43)	8.02 (7.33–8.68)	9.22 (8.47–9.93)	9.56 (8.81–10.27)	0.80
IDI (by sex)	8.82 (8.07–9.53)	8.44 (7.61–9.22)	9.51 (8.80–10.19)	9.82 (9.11–10.51)	1.00
IDI (by age cut-off & sex)	8.70 (8.06–9.30)	8.61 (7.91–9.27)	9.61 (8.83–10.34)	9.50 (8.70–10.26)	0.80
IDI (by age-groups)	8.79 (8.11–9.44)	8.15 (7.48–8.79)	8.84 (8.13–9.53)	9.58 (8.79–10.33)	0.79
PHI (overall)	8.38 (7.62–9.09)	9.00 (8.17–9.77)	9.68 (8.95–10.37)	9.64 (8.94–10.30)	1.25
PHI (by age cut-off)	8.43 (7.75–9.07)	8.23 (7.52–8.90)	9.79 (9.04–10.51)	9.92 (9.15–10.65)	1.49
Females					
Life expectancy in the general population					
IDI (overall)	24.54	24.20	26.41	26.79	2.25
IDI (by age cut-off)	24.60	24.72	26.35	26.80	2.20
IDI (by sex)	24.78	23.74	25.76	26.74	1.97
IDI (by age cut-off & sex)	24.69	24.61	25.39	26.76	2.07
IDI (by age-groups)	24.56	24.83	26.45	26.94	2.38
PHI (overall)	23.35	25.00	26.38	27.23	3.88
PHI (by age cut-off)	23.52	24.98	26.51	27.23	3.71
Life expectancy in the cancer population					
IDI (overall)	15.17 (14.33–16.07)	15.03 (14.19–15.92)	16.35 (15.60–17.14)	17.59 (16.83–18.40)	2.42
IDI (by age cut-off)	14.69 (13.97–15.45)	16.24 (15.55–16.95)	16.60 (15.76–17.48)	17.39 (16.51–18.33)	2.71
IDI (by sex)	15.48 (14.58–16.44)	14.81 (13.95–15.72)	15.86 (15.09–16.67)	17.33 (16.65–18.04)	1.85
IDI (by age cut-off & sex)	15.08 (14.33–15.88)	15.95 (15.24–16.69)	15.78 (14.99–16.62)	17.37 (16.57–18.21)	2.29
IDI (by age-groups)	14.78 (14.07–15.51)	16.23 (15.55–16.94)	17.33 (16.51–18.18)	17.61 (16.66–18.62)	2.84
PHI (overall)	14.40 (13.58–15.27)	15.23 (14.35–16.17)	16.47 (15.71–17.26)	17.34 (16.59–18.12)	2.94
PHI (by age cut-off)	14.45 (13.71–15.24)	15.93 (15.19–16.71)	16.24 (15.42–17.10)	17.57 (16.74–18.44)	3.11
Loss in life expectancy					
IDI (overall)	9.36 (8.47–10.21)	9.17 (8.28–10.01)	10.06 (9.27–10.81)	9.19 (8.39–9.96)	−0.17
IDI (by age cut-off)	9.91 (9.15–10.63)	8.49 (7.77–9.17)	9.75 (8.87–10.59)	9.40 (8.47–10.29)	−0.51
IDI (by sex)	9.29 (8.34–10.19)	8.93 (8.02–9.79)	9.90 (9.09–10.67)	9.42 (8.71–10.10)	0.12
IDI (by age cut-off & sex)	9.61 (8.81–10.37)	8.66 (7.91–9.37)	9.61 (8.77–10.40)	9.39 (8.55–10.19)	−0.22
IDI (by age-groups)	9.79 (9.05–10.49)	8.60 (7.89–9.28)	9.13 (8.27–9.94)	9.33 (8.32–10.28)	−0.46
PHI (overall)	8.95 (8.08–9.77)	9.76 (8.83–10.65)	9.91 (9.12–10.67)	9.89 (9.11–10.64)	0.94
PHI (by age cut-off)	9.07 (8.29–9.81)	9.04 (8.27–9.79)	10.27 (9.41–11.09)	9.66 (8.79–10.49)	0.59

*IDI* individual disposable income, *PHI* part of household income, *SEP* socioeconomic position

**Table 3:** Life expectancy with and without colon cancer (in years) for a 75-year-old individual and loss in life expectancy after the cancer diagnosis by income group (from the lowest (Q1) to the highest (Q4) income) and across different ways to create the income quartiles, by sex. The difference between the highest (Q4) and lowest (Q1) income groups is also provided. 95% confidence intervals are given in the parentheses

Indicator for SEP	Q1	Q2	Q3	Q4	Difference Q4-Q1
Males					
Life expectancy in the general population					
IDI (overall)	9.84	10.18	11.36	12.22	2.38
IDI (by age cut-off)	9.89	10.07	10.99	12.02	2.13
IDI (by sex)	9.92	10.61	11.80	12.23	2.31
IDI (by age cut-off & sex)	9.89	10.42	11.53	12.22	2.33
IDI (by age-groups)	9.87	10.15	10.81	11.99	2.12
PHI (overall)	9.71	10.39	11.58	12.23	2.52
PHI (by age cut-off)	9.70	10.22	11.32	12.22	2.51
Life expectancy in the cancer population					
IDI (overall)	6.43 (6.17–6.70)	6.93 (6.71–7.15)	7.81 (7.58–8.06)	8.49 (8.20–8.79)	2.06
IDI (by age cut-off)	6.68 (6.42–6.94)	6.68 (6.45–6.92)	7.57 (7.35–7.80)	8.37 (8.11–8.65)	1.69
IDI (by sex)	6.55 (6.33–6.77)	7.21 (6.99–7.43)	8.34 (8.08–8.61)	8.51 (8.20–8.83)	1.96
IDI (by age cut-off & sex)	6.63 (6.41–6.85)	7.04 (6.82–7.26)	7.91 (7.66–8.17)	8.56 (8.27–8.85)	1.93
IDI (by age-groups)	6.66 (6.40–6.93)	6.76 (6.53–6.99)	7.37 (7.15–7.60)	8.39 (8.14–8.65)	1.73
PHI (overall)	6.42 (6.19–6.66)	7.04 (6.82–7.26)	7.91 (7.65–8.17)	8.64 (8.35–8.93)	2.22
PHI (by age cut-off)	6.44 (6.21–6.68)	7.02 (6.80–7.25)	7.67 (7.43–7.92)	8.49 (8.21–8.77)	2.05
Loss in life expectancy					
IDI (overall)	3.42 (3.15–3.68)	3.25 (3.03–3.47)	3.55 (3.31–3.78)	3.73 (3.43–4.03)	0.31
IDI (by age cut-off)	3.21 (2.94–3.46)	3.39 (3.15–3.62)	3.42 (3.20–3.64)	3.65 (3.38–3.91)	0.44
IDI (by sex)	3.37 (3.14–3.59)	3.40 (3.18–3.62)	3.46 (3.19–3.72)	3.72 (3.39–4.03)	0.35
IDI (by age cut-off & sex)	3.26 (3.04–3.48)	3.38 (3.16–3.60)	3.62 (3.36–3.88)	3.66 (3.37–3.95)	0.40
IDI (by age-groups)	3.21 (2.94–3.46)	3.39 (3.16–3.62)	3.44 (3.21–3.66)	3.60 (3.35–3.85)	0.39
PHI (overall)	3.28 (3.04–3.52)	3.35 (3.12–3.57)	3.67 (3.41–3.93)	3.59 (3.29–3.88)	0.30
PHI (by age cut-off)	3.27 (3.03–3.50)	3.20 (2.97–3.42)	3.65 (3.41–3.89)	3.73 (3.45–4.00)	0.46
Females					
Life expectancy in the general population					
IDI (overall)	12.75	12.59	13.74	13.63	0.88
IDI (by age cut-off)	12.81	12.44	13.63	13.61	0.80
IDI (by sex)	12.94	12.26	13.47	13.69	0.75
IDI (by age cut-off & sex)	12.98	12.34	12.95	13.69	0.72
IDI (by age-groups)	12.88	12.36	13.46	13.69	0.81
PHI (overall)	12.13	13.12	13.84	14.01	1.88
PHI (by age cut-off)	12.13	12.89	13.78	14.00	1.87
Life expectancy in the cancer population					
IDI (overall)	8.89 (8.65–9.13)	8.77 (8.51–9.04)	9.55 (9.23–9.89)	9.69 (9.31–10.08)	0.80
IDI (by age cut-off)	8.94 (8.70–9.19)	8.50 (8.24–8.77)	9.51 (9.20–9.84)	9.72 (9.37–10.09)	0.78
IDI (by sex)	9.12 (8.85–9.39)	8.38 (8.12–8.64)	9.41 (9.11–9.71)	9.74 (9.39–10.10)	0.62
IDI (by age cut-off & sex)	9.03 (8.76–9.31)	8.54 (8.28–8.81)	8.94 (8.66–9.24)	9.70 (9.39–10.03)	0.67
IDI (by age-groups)	9.02 (8.78–9.27)	8.45 (8.19–8.72)	9.35 (9.04–9.67)	9.84 (9.49–10.20)	0.82
PHI (overall)	8.38 (8.13–8.63)	9.04 (8.77–9.32)	9.69 (9.38–10.02)	9.97 (9.62–10.33)	1.59
PHI (by age cut-off)	8.34 (8.09–8.60)	8.92 (8.65–9.19)	9.45 (9.14–9.77)	10.04 (9.71–10.38)	1.70
Loss in life expectancy					
IDI (overall)	3.86 (3.61–4.09)	3.82 (3.55–4.08)	4.19 (3.85–4.52)	3.94 (3.55–4.31)	0.08
IDI (by age cut-off)	3.87 (3.62–4.11)	3.94 (3.67–4.20)	4.12 (3.79–4.43)	3.89 (3.53–4.24)	0.02
IDI (by sex)	3.82 (3.55–4.09)	3.88 (3.61–4.14)	4.06 (3.76–4.36)	3.96 (3.60–4.30)	0.13
IDI (by age cut-off & sex)	3.94 (3.66–4.21)	3.80 (3.53–4.06)	4.01 (3.71–4.29)	3.99 (3.67–4.30)	0.05
IDI (by age-groups)	3.85 (3.60–4.10)	3.91 (3.64–4.17)	4.11 (3.79–4.42)	3.85 (3.49–4.20)	−0.01
PHI (overall)	3.75 (3.50–4.00)	4.08 (3.79–4.35)	4.15 (3.82–4.46)	4.05 (3.68–4.40)	0.29
PHI (by age cut-off)	3.79 (3.54–4.04)	3.98 (3.70–4.25)	4.33 (4.01–4.64)	3.96 (3.62–4.29)	0.17

*IDI* individual disposable income, *PHI* part of household income, *SEP* socioeconomic position

### Defining SEP by education

When the highest attained education was used as an indicator for SEP, smaller disparities were observed for standardised estimates (Table 4). Estimates for age- and sex-specific measures can also be found in Supplementary Tables S5, S6.

**Table 4:** Age and sex standardised estimates of relative survival (%) and life expectancy (in years) by education group as well as the difference between the highest and lowest education levels. 95% confidence intervals are given in the parentheses

Estimate	Years after diagnosis	Completed < 9 years of compulsory education	Completed 9 years of compulsory education	Secondary education	Tertiary education	Difference Highest -Lowest
Relative survival	1	82 (81–83)	83 (81–84)	84 (84–0.85)	85 (84–86)	3
Relative survival	5	65 (64–67)	66 (64–69)	67 (0.66–0.68)	69 (67–70)	3
Relative survival	10	61 (59–63)	60 (57–64)	63 (0.61–0.64)	64 (62–66)	3
Life expectancy in the general population		14.07	14.80	14.99	16.22	2.15
Life expectancy in the cancer population		9.02 (8.54–9.53)	8.92 (8.48–9.38)	9.68 (9.46–9.91)	10.61 (10.31–10.91)	1.59
Loss in life expectancy		5.05 (4.54–5.53)	5.88 (5.42–6.32)	5.30 (5.08–5.52)	5.62 (5.31–5.91)	0.57

## Discussion

We have explored how sensitive estimates of relative survival and life expectancy metrics are to the definition of SEP using data on Swedish colon cancer patients. We utilised IDI, PHI and highest attained education as indicators of SEP and allocated individuals in groups of high and low SEP. For income, income categories were created based on all patients at once, or separately by sex or age group, or both. We showed that standardised estimates yield small variations across the different ways used to create income groups. However, we found larger variation for age-specific estimates, especially for younger individuals; the disparities in relative survival between the SEP groups varied up to 4% points across SEP definitions while disparities in LLE showed e.g., for 60-year-old females, 0.94 years higher loss for the highest income group under one SEP definition (PHI overall) but 0.46 years lower loss under another definition (IDI by age-groups).

The variation observed in the estimated disparities across SEP definition is particularly important for studies exploring LLE differences by SEP groups. For instance, a previous study on English colorectal cancer data [6], found that 60-year-old males from the least deprived group lost 9.54 years on average due to their colon cancer diagnosis while similar males from the most deprived group lost 9.04 years i.e. 0.5 years less (6 months). Such differences between SEP groups is partly driven by the way that SEP is measured and other conceptualisations of SEP may have resulted in disparities of a different magnitude.

Our study contributes to the current literature on disparities in prognosis after a cancer diagnosis by socioeconomic groups, particularly the lack of discussion on the indicators used to conceptualise SEP. A key strength of our study is the rich and large data resource on colon cancer, which includes information on several socioeconomic variables, allowing us to compare various individual-level indicators of income and education. We also addressed potential misclassifications by creating quartiles separately for specific groups. Additionally, we went beyond survival probabilities and evaluated disparities in life expectancy measures, which are becoming increasingly popular in cancer epidemiology. Despite the comprehensive definitions of SEP considered in our study, there are other ways of defining SEP that we did not include in our analysis, such as area-level indicators. However, area-level indicators have been shown to not be sufficient for replacing individual-level variation. Moreover, our analysis showed that relative survival and LLE estimates are influenced by the way that SEP is created, especially for age-specific estimates, however, it is not possible to identify a single superior definition, as the optimal definition depends on the underlying mechanisms of SEP disparities and the population dynamics under study.

Different indicators represent different mechanisms but are usually strongly correlated. Careful consideration should be given to the most relevant indicator for each study. Education reflects receptiveness to health education messages and the ability to communicate with and access health services [22, 40]. Household income should be chosen if interest is on material resources or the purchasing powers of households, while individual income should be used if an individual’s status and socioeconomic standing is to be measured [27]. In certain cases household based measures cannot be replaced by individual measures, as the latter may be not a good indicator for the SEP of those with e.g. low individual income but access to other income sources.

Using IDI as an indicator for SEP, can be problematic for older individuals, who will be allocated to the lowest SEP more often than the working population. Many of these individuals would have been in a higher SEP prior to their retirement. As the retirement changes are not likely to influence to a large extent the individual’s position in society, this group of patients will be misclassified to a lower SEP if IDI after retirement is used to define SEP. Creating separate quartiles for patients above 65 years enables a comparison within a group of retired individuals and yield a more appropriate allocation in SEP groups. However, the age of retirement is not fixed and that is why smaller age groups were also considered in our study. These are also relevant for the youngest who may have a lower income due to for example studies or parental leave. IDI may also be problematic to use in elderly women. Housewives will have a low IDI but may be part of a household with more resources. In these situations, we believe that household-based income indicators, are more appropriate since they represent the availability of material resources and corresponding health awareness. Further attempts to create income groups based on disposable income per consumption unit for family separately by a cut-off of 65 years old had no impact on the estimates. This way of addressing potential misclassification of individuals into lower SEP by creating quartiles separately by age or sex can, in principle, be extended to other factors if certain characteristics are believed to cause misclassification when ignored. However, we must carefully evaluate how these factors influence SEP in the studied population, as this may vary by population, e.g. by country. If the allocation of a group with a certain characteristic to lower SEP groups is believed to be an appropriate reflection of their SEP, constructing separate quartiles by this characteristic could lead to an underestimation of actual disparities.

A few studies have investigated how different conceptualisations of SEP influence socioeconomic disparities, with findings suggesting that varying SEP definitions result in disparities of different magnitudes, as observed in our study. One study in England and Wales focused on colorectal cancer and found that both individual-level indicators (such as education, occupation, and housing tenure) and area-based indicators of deprivation were associated with survival [41]. Another study examined the relationship between cancer survival and various SEP indicators, both area-based and individual-level (including occupation, education, and income) [2]. This study identified inequalities in colorectal cancer survival at both levels but did not find evidence of contextual effect modification of individual-level socioeconomic inequalities across different area-level contexts. In Finland, research on educational differences in colorectal cancer survival suggested that area-based indicators are not suitable substitutes for individual-level indicators [42]. Additionally, a study in Canada and the US emphasized the importance of carefully selecting SEP indicators, as the magnitude of disparities depends on the chosen indicators. However, previous studies primarily reported hazard ratios and survival measures, with limited research on how the selection of the SEP indicator affects life expectancy metrics [43].

## Conclusions

Exploring how cancer prognosis varies by SEP is of high importance. In this way disparities can be quantified and the groups with worse prognosis can be targeted with relevant policies to reduce disparities. An essential part of cancer disparities studies is to carefully consider the indicators used to conceptualise SEP. In our analysis, different definitions led to large variation on the estimated disparities, especially for age-specific measures. The choice of how to conceptualise SEP should be based not on the magnitude of the estimated disparities but on what indicator best reflects the SEP pathway that is of interest.

## Supplementary Information

Supplementary Material 1.

## Data Availability

This study analysed data from the Colorectal Cancer Database Sweden (CRCBaSe). Restrictions apply to the availability of these data, and so are not publicly available. However, data requests can be send to the relevant quality registry for colorectal cancer https://cancercentrum.se/samverkan/cancerdiagnoser/tjocktarm-andtarm-och-anal/tjock--och-andtarm/kvalitetsregister/forskning/forskningsdatabas/.

## Abbreviations

CRCBaSe: Colorectal cancer database
IDI: Individual disposable income
LE: Life expectancy
LLE: Loss in life expectancy
PHI: Part of household income
SEP: Socioeconomic position
