# Supplementary material for: Loss in life expectancy after a colon cancer diagnosis by socioeconomic group: does the indicator of socioeconomic position matter?
Source: BMC Public Health. 2025 Nov 28;25:4282. doi: 10.1186/s12889-025-25610-y (PMC12723879; doi:10.1186/s12889-025-25610-y)
Supplement: Supplementary file 1 — Supplementary Material 1. [file 12889_2025_25610_MOESM1_ESM.pdf]

A) Supplementary tables and figure

Table S1: Number of colon cancer patients in each income group (Q1-Q4) when individual disposable income is used as an indicator and quartiles are created based on all patients (overall) compared to different indicators and ways to create the quartiles. IDI=individual disposable income, PHI=part of household income, SEP=socioeconomic position

Other indicators for SEP		IDI (overall)			
		Q1	Q2	Q3	Q4
IDI (by age cut-off)	Q1	8,591	911	250	0
	Q2	1,043	6,658	2,210	0
	Q3	0	2,504	5,618	2,048
	Q4	0	0	2,110	7,583
IDI (by sex)	Q1	7,598	1,920	0	0
	Q2	2,036	5,692	2,492	0
	Q3	0	2,461	6,140	1,550
	Q4	0	0	1,556	8,081
IDI (by age cut-off & sex)	Q1	7,219	2,181	288	0
	Q2	2,415	4,494	2,747	378
	Q3	0	3,398	4,914	1,713
	Q4	0	0	2,239	7,540
IDI (by age groups)	Q1	8,166	1,334	308	0
	Q2	1,468	5,399	2,900	94
	Q3	0	3,340	4,071	2,614
	Q4	0	0	2,909	6,923
PHI (overall)	Q1	5,912	3,080	277	87
	Q2	2,217	5,205	2,674	276
	Q3	1,146	1,421	5,686	1,865
	Q4	359	367	1,551	7,403
PHI (by age cut-off)	Q1	5,824	2,794	545	273
	Q2	2,092	5,157	2,307	680
	Q3	1,266	1,614	5,477	1,812
	Q4	452	508	1,859	6,866

Table S2: One, five and ten-year age and sex standardised relative survival (%) by income groups (Q1-Q4) across different ways used to create income quartiles as an indicator for socioeconomic position (SEP) as well as the difference in age and sex standardised survival between the highest (Q4) and lowest (Q1) income groups. 95% confidence intervals are given in the parentheses. IDI=individual disposable income, PHI=part of household income, SEP=socioeconomic position

Years After Diagnosis	Indicator for SEP	Q1	Q2	Q3	Q4	Difference Q4-Q1
1	IDI (overall)	81 (80 - 82)	81 (81 - 82)	84 (83 - 85)	86 (85 - 87)	5
1	IDI (by age cut-off)	81 (81 - 82)	82 (81 - 83)	84 (83 - 85)	86 (85 - 87)	4
1	IDI (by sex)	82 (81 - 83)	82 (81 - 83)	84 (83 - 85)	86 (85 - 86)	4
1	IDI (by age cut-off & sex)	81 (81 - 82)	82 (82 - 83)	84 (83 - 85)	86 (85 - 86)	4
1	IDI (by age-groups)	82 (81 - 83)	82 (81 - 83)	83 (83 - 84)	86 (85 - 86)	4
1	PHI (overall)	80 (79 - 81)	83 (82 - 83)	85 (84 - 85)	86 (85 - 87)	6
1	PHI (by age cut-off)	81 (80 - 81)	83 (82 - 83)	84 (83 - 85)	86 (85 - 87)	5
5	IDI (overall)	65 (63 - 66)	65 (64 - 67)	67 (65 - 68)	69 (67 - 71)	4
5	IDI (by age cut-off)	65 (64 - 66)	66 (64 - 67)	66 (65 - 68)	69 (67 - 70)	4
5	IDI (by sex)	65 (64 - 67)	65 (64 - 67)	68 (67 - 69)	68 (67 - 70)	3
5	IDI (by age cut-off & sex)	65 (64 - 66)	66 (65 - 67)	66 (65 - 68)	69 (68 - 70)	4
5	IDI (by age-groups)	65 (64 - 67)	65 (64 - 67)	67 (65 - 68)	69 (68 - 70)	4
5	PHI (overall)	64 (62 - 65)	66 (65 - 67)	67 (66 - 69)	69 (67 - 70)	5
5	PHI (by age cut-off)	64 (63 - 66)	67 (66 - 68)	66 (65 - 68)	69 (67 - 70)	4
10	IDI (overall)	59 (57 - 62)	61 (59 - 64)	61 (59 - 63)	64 (62 - 66)	4
10	IDI (by age cut-off)	60 (58 - 62)	62 (59 - 64)	62 (59 - 64)	64 (61 - 66)	3
10	IDI (by sex)	60 (58 - 62)	61 (59 - 63)	63 (61 - 65)	64 (61 - 66)	3
10	IDI (by age cut-off & sex)	60 (58 - 63)	61 (59 - 63)	62 (60 - 64)	64 (62 - 66)	3
10	IDI (by age-groups)	60 (58 - 63)	61 (59 - 63)	62 (60 - 64)	64 (62 - 66)	3
10	PHI (overall)	60 (58 - 63)	61 (59 - 63)	62 (60 - 64)	64 (62 - 66)	4
10	PHI (by age cut-off)	60 (58 - 63)	63 (61 - 65)	61 (59 - 63)	64 (61 - 66)	3

Table S3: Age and sex standardised life expectancy with and without colon cancer (in years) and loss in life expectancy after a cancer diagnosis by income groups (Q1-Q4) across different ways used to create income quartiles as an indicator for socioeconomic position (SEP) as well as the difference between the highest (Q4) and lowest (Q1) income groups. 95% confidence intervals are given in the parentheses. IDI=individual disposable income, PHI=part of household income, SEP=socioeconomic position

Indicator for SEP	Q1	Q2	Q3	Q4	Difference Q4-Q1
		Life expectancy in the general population			
IDI (overall)	13.93	13.94	15.44	15.98	2.05
IDI (by age cut-off)	13.99	14.16	15.35	15.93	1.94
IDI (by sex)	14.03	14.10	15.55	16.03	2.00
IDI (by age cut-off & sex)	14.05	14.36	15.29	16.06	2.00
IDI (by age-groups)	13.99	14.23	15.24	15.99	2.00
PHI (overall)	13.50	14.51	15.62	16.18	2.67
PHI (by age cut-off)	13.60	14.45	15.57	16.18	2.59
		Life expectancy in the cancer population			
IDI (overall)	8.70 (8.38 - 9.02)	8.86 (8.54 - 9.19)	9.77 (9.49 - 10.05)	10.46 (10.17 - 10.75)	1.76
IDI (by age cut-off)	8.75 (8.48 - 9.02)	9.07 (8.81 - 9.34)	9.81 (9.50 - 10.12)	10.36 (10.04 - 10.69)	1.61
IDI (by sex)	8.84 (8.53 - 9.16)	8.92 (8.59 - 9.27)	9.95 (9.67 - 10.24)	10.42 (10.15 - 10.71)	1.59
IDI (by age cut-off & sex)	8.82 (8.56 - 9.09)	9.20 (8.94 - 9.47)	9.74 (9.44 - 10.05)	10.46 (10.15 - 10.79)	1.64
IDI (by age-groups)	8.77 (8.52 - 9.03)	9.08 (8.82 - 9.34)	9.84 (9.54 - 10.14)	10.46 (10.12 - 10.81)	1.69
PHI (overall)	8.48 (8.19 - 8.77)	9.17 (8.86 - 9.49)	9.97 (9.69 - 10.26)	10.45 (10.16 - 10.75)	1.98
PHI (by age cut-off)	8.55 (8.30 - 8.82)	9.36 (9.09 - 9.64)	9.75 (9.45 - 10.07)	10.37 (10.03 - 10.71)	1.82
		Loss in life expectancy			
IDI (overall)	5.24 (4.91 - 5.55)	5.08 (4.75 - 5.40)	5.67 (5.38 - 5.95)	5.53 (5.24 - 5.81)	0.29
IDI (by age cut-off)	5.24 (4.97 - 5.51)	5.10 (4.83 - 5.36)	5.54 (5.23 - 5.84)	5.57 (5.23 - 5.89)	0.32
IDI (by sex)	5.19 (4.87 - 5.50)	5.17 (4.83 - 5.51)	5.61 (5.32 - 5.88)	5.61 (5.32 - 5.88)	0.41
IDI (by age cut-off & sex)	5.23 (4.96 - 5.50)	5.16 (4.89 - 5.42)	5.55 (5.24 - 5.85)	5.59 (5.27 - 5.91)	0.36
IDI (by age-groups)	5.22 (4.96 - 5.48)	5.15 (4.89 - 5.41)	5.40 (5.10 - 5.70)	5.53 (5.18 - 5.88)	0.31
PHI (overall)	5.03 (4.73 - 5.32)	5.34 (5.02 - 5.65)	5.64 (5.35 - 5.92)	5.73 (5.43 - 6.02)	0.70
PHI (by age cut-off)	5.05 (4.78 - 5.30)	5.09 (4.81 - 5.36)	5.82 (5.50 - 6.12)	5.82 (5.47 - 6.15)	0.77

Table S4: Relative survival for a patient diagnosed with colon cancer at 75 years old by income group (from the lowest (Q1) to the highest (Q4) income) and across different ways to create the income quartiles, by sex. The difference between the highest (Q4) and lowest (Q1) income groups is also provided. 95% confidence intervals are given in the parentheses. IDI=individual disposable income, PHI=part of household income, SEP=socioeconomic position

Years After Diagnosis	Indicator for SEP	Q1	Q2	Q3	Q4	Difference Q4-Q1
**Males**						
1	IDI (overall)	81 (79 - 82)	82 (81 - 83)	85 (84 - 86)	86 (85 - 87)	5
1	IDI (by age cut-off)	82 (80 - 83)	81 (79 - 82)	84 (83 - 86)	86 (85 - 87)	4
1	IDI (by sex)	81 (80 - 83)	83 (82 - 84)	86 (85 - 87)	86 (85 - 87)	5
1	IDI (by age cut-off & sex)	82 (80 - 83)	83 (82 - 84)	85 (84 - 86)	86 (85 - 88)	5
1	IDI (by age-groups)	82 (80 - 84)	82 (80 - 83)	84 (82 - 85)	86 (85 - 87)	4
1	PHI (overall)	80 (79 - 82)	83 (82 - 84)	85 (84 - 86)	87 (85 - 88)	6
1	PHI (by age cut-off)	80 (79 - 82)	83 (81 - 84)	85 (84 - 86)	86 (85 - 87)	6
5	IDI (overall)	64 (61 - 67)	66 (64 - 68)	69 (67 - 71)	70 (67 - 72)	6
5	IDI (by age cut-off)	66 (64 - 69)	65 (62 -.67)	68 (66 - 70)	70 (68 - 72)	4
5	IDI (by sex)	65 (63 - 67)	67 (65 - 69)	71 (69 - 73)	70 (67 - 72)	5
5	IDI (by age cut-off & sex)	66 (63 - 68)	66 (64 - 68)	68 (66 - 70)	70 (68 - 72)	5
5	IDI (by age-groups)	66 (64 - 69)	65 (63 - 67)	67 (65 - 69)	70 (68 - 72)	4
5	PHI (overall)	64 (62 - 67)	67 (65 - 69)	68 (66 - 70)	71 (68 - 73)	7
5	PHI (by age cut-off)	65 (62 - 67)	67 (65 - 69)	67 (65 - 69)	70 (68 - 72)	5
10	IDI (overall)	58 (54 - 62)	62 (59 - 65)	62 (60 - 65)	64 (61 - 67)	6
10	IDI (by age cut-off)	61 (58 - 65)	60 (57 - 63)	62 (60 - 65)	64 (61 - 67)	3
10	IDI (by sex)	59 (56 - 62)	62 (59 - 65)	65 (62 - 68)	64 (61 - 67)	5
10	IDI (by age cut-off & sex)	60 (57 - 64)	61 (58 - 64)	63 (60 - 66)	64 (61 - 67)	4
10	IDI (by age-groups)	61 (58 - 65)	60 (57 - 63)	62 (59 - 65)	64 (62 - 67)	4
10	PHI (overall)	60 (57 - 63)	61 (58 - 64)	62 (59 - 65)	65 (62 - 68)	5
10	PHI (by age cut-off)	60 (57 - 63)	62 (60 - 66)	61 (58 - 64)	64 (61 - 67)	4
**Females**						
1	IDI (overall)	84 (83 - 85)	83 (81 - 84)	85 (84 - 86)	86 (85 - 88)	3
1	IDI (by age cut-off)	83 (82 -.84)	82 (81 - 83)	85 (84 - 86)	87 (85 - 88)	3
1	IDI (by sex)	84 (83 - 85)	82 (81 - 83)	84 (83 - 86)	86 (85 - 88)	2
1	IDI (by age cut-off & sex)	83 (82 - 84)	83 (82 - 84)	84 (83 - 85)	86 (85 - 87)	3
1	IDI (by age-groups)	84 (83 - 85)	82 (81 - 84)	84 (83 - 86)	87 (85 - 88)	3
1	PHI (overall)	82 (80 - 83)	83 (82 - 85)	86 (84 - 87)	86 (85 - 88)	5
1	PHI (by age cut-off)	82 (81 - 83)	83 (82 - 84)	85 (84 - 86)	87 (85 - 88)	5
5	IDI (overall)	69 (67 - 71)	68 (66 - 70)	70 (67 - 72)	71 (68 - 73)	2
5	IDI (by age cut-off)	69 (67 - 71)	67 (65 - 69)	69 (67 - 72)	71 (69 - 74)	2
5	IDI (by sex)	70 (68 - 72)	67 (65 - 69)	70 (68 - 72)	71 (68 - 73)	1
5	IDI (by age cut-off & sex)	69 (67 - 71)	68 (66 - 70)	68 (66 - 70)	71 (69 - 73)	2
5	IDI (by age-groups)	69 (68 - 71)	67 (65 - 69)	69 (67 - 71)	72 (69 - 74)	2

Years After Diagnosis	Indicator for SEP	Q1	Q2	Q3	Q4	Difference Q4-Q1
5	PHI (overall)	67 (65 - 69)	69 (67 - 71)	70 (68 - 72)	71 (69 - 74)	4
5	PHI (by age cut-off)	67 (65 - 69)	68 (66 - 70)	69 (67 - 71)	72 (69 - 74)	4
10	IDI (overall)	66 (63 - 68)	66 (63 - 69)	65 (62 - 68)	67 (64 - 70)	1
10	IDI (by age cut-off)	66 (63 - 68)	64 (61 - 67)	66 (63 - 69)	67 (64 - 70)	1
10	IDI (by sex)	67 (64 - 69)	64 (61 - 67)	66 (63 - 69)	67 (64 - 70)	1
10	IDI (by age cut-off & sex)	66 (63 - 68)	65 (62 - 67)	65 (62 - 67)	67 (64 - 70)	1
10	IDI (by age-groups)	66 (64 - 68)	64 (61 - 67)	65 (63 - 68)	68 (65 - 71)	2
10	PHI (overall)	65 (62 - 68)	65 (62 - 68)	66 (63 - 69)	67 (64 - 70)	2
10	PHI (by age cut-off)	65 (62 -.67)	65 (63 - 68)	64 (62 - 67)	68 (65 - 71)	3

Table S5: : One, five and ten-year relative survival (%)and life expectancy (in years) for a 60-year-old individual by education group as well as the difference between the highest and lowest education levels, by sex. 95% confidence intervals are given in the parentheses.

Estimate	Years after diagnosis	Completed <9 years of compulsory education	Completed 9 years of compulsory education	Secondary education	Tertiary education	Difference Highest-Lowest
**Males**						
Relative survival	1	85 (84 - 87)	84 (82 - 86)	87 (86 - 88)	88 (87 - 89)	3
Relative survival	5	66 (62 - 69)	63 (60 - 67)	67 (65 - 68)	69 (67 - 71)	3
Relative survival	10	59 (55 - 63)	55 (50 - 59)	60 (58 - 62)	62 (59 - 65)	3
Life expectancy in the general population		21.27	21.93	22.84	24.80	3.53
Life expectancy in the cancer population		12.93 (12.12 - 13.79)	12.19 (11.23 - 13.24)	13.89 (13.33 - 14.47)	15.39 (14.65 - 16.16)	2.46
Loss in life expectancy		8.35 (7.48 - 9.16)	9.74 (8.69 - 10.70)	8.95 (8.37 - 9.51)	9.41 (8.64 - 10.15)	1.06
**Females**						
Relative survival	1	86 (84 - 88)	0.83 (81 - 85)	86 (85 - 87)	89 (88 - 90)	3
Relative survival	5	68 (65 - 71)	0.62 (59 - 66)	66 (65 - 68)	72 (70 - 74)	4
Relative survival	10	64 (60 - 68)	0.56 (51 - 61)	62 (59 - 64)	68 (65 - 71)	4
Life expectancy in the general population		23.98	25.33	25.38	27.36	3.38
Life expectancy in the cancer population		15.54 (14.62 - 16.51)	14.07 (12.86 - 15.39)	15.82 (15.21 - 16.46)	18.42 (17.66 – 19.21)	2.88
Loss in life expectancy		8.44 (7.47 - 9.35)	11.26 (9.94 - 12.47)	9.56 (8.92 - 10.17)	8.94 (8.15 – 9.70)	0.50

Table S6: One, five and ten-year relative survival (%) and life expectancy (in years) for a 75-year-old individual by education group as well as the difference between the highest and lowest education levels, by sex. 95% confidence intervals are given in the parentheses.

Estimate	Years after diagnosis	Completed <9 years of compulsory education	Completed 9 years of compulsory education	Secondary education	Tertiary education	Difference Highest-Lowest
**Males**						
Relative survival	1	82 (81 - 84)	83 (80 - 85)	85 (84 - 86)	85 (84 - 86)	3
Relative survival	5	66 (64 - 68)	67 (63 - 71)	69 (67 - 71)	68 (66 - 71)	2
Relative survival	10	61 (58 - 64)	60 (55 - 66)	64 (61 - 66)	63 (60 - 66)	2
Life expectancy in the general population		10.29	10.69	11.01	12.14	1.85
Life expectancy in the cancer population		6.93 (6.73 - 7.13)	7.19 (6.75 - 7.67)	7.67 (7.47 - 7.88)	8.29 (7.99 - 8.59)	1.36
Loss in life expectancy		3.36 (3.15 - 3.56)	3.49 (3.02 - 3.94)	3.34 (3.13 - 3.54)	3.85 (3.55 - 4.14)	0.49
**Females**						
Relative survival	1	83 (82 - 84)	81 (79 - 84)	84 (83 - 85)	86 (85 - 87)	3
Relative survival	5	68 (66 - 70)	66 (63 - 70)	69 (67 - 70)	72 (69 - 74)	4
Relative survival	10	65 (63 - 68)	61 (56 - 66)	65 (63 - 68)	68 (65 - 71)	3
Life expectancy in the general population		12.29	13.43	13.11	14.31	2.02
Life expectancy in the cancer population		8.55 (8.31 - 8.79)	8.80 (8.28 - 9.35)	9.11 (8.87 - 9.35)	10.23 (9.89 – 10.58)	1.68
Loss in life expectancy		3.74 (3.50 - 3.98)	4.63 (4.08 - 5.15)	4.00 (3.75 - 4.24)	4.08 (3.73 – 4.14)	0.34

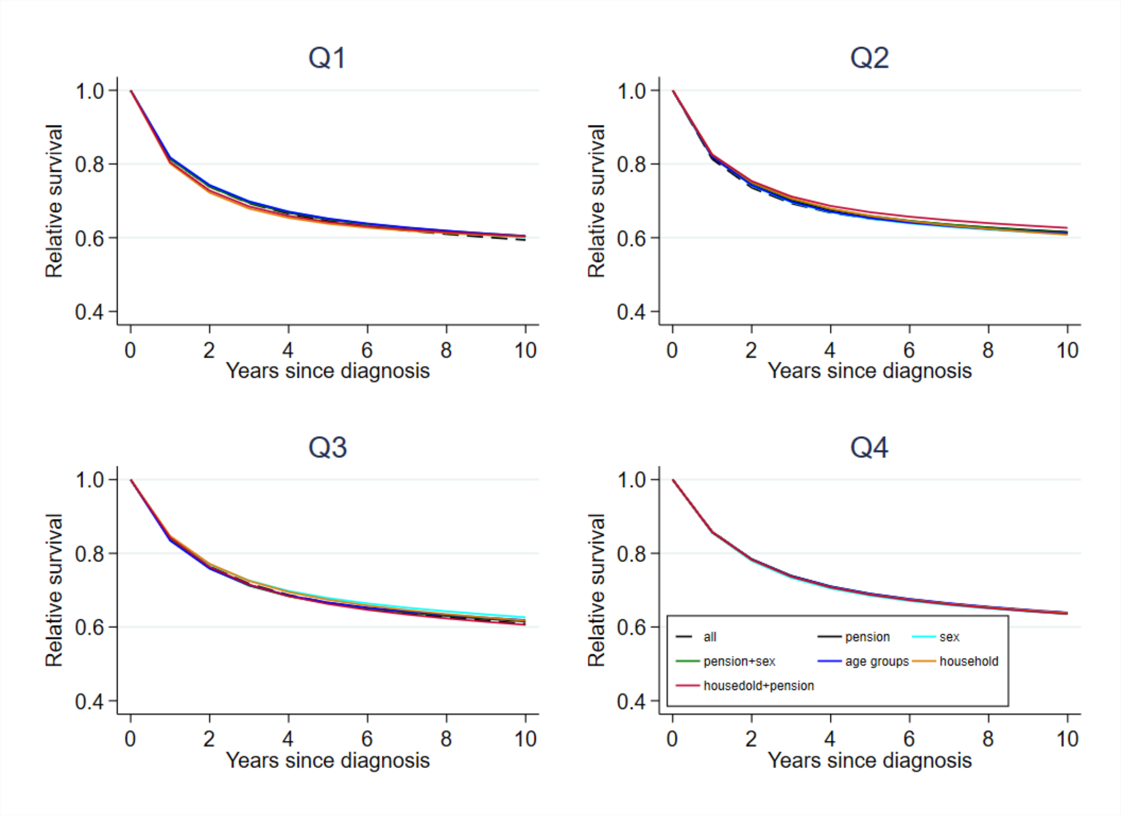

Figure S1: Age and sex standardised relative survival (%) by income group (from the lowest (Q1) to the highest (Q4) income) and across different ways to create the income quartiles. The legends correspond to the following ways to create quartiles: all= using individual disposable income (IDI) overall, pension=IDI and separately above and below 65 years old, sex=IDI and separately for males and females, pension+sex= IDI and separately by sex and age cut-off 65 years, age-groups= IDI and separately by age-groups, household= using part of household income (PHI) overall, household+pension= PHI and separately by age cut-off of 65 years.

B) Cut-offs

The cut-offs used to create the income groups for the cancer population is provided. The cut-offs are based on the quartiles of the income distribution of the control population that was available in the data (income in 100s of SEK.).

Indicator: individual disposable income

i - quantiles based on all individuals (overall)
Cut-offs: min, 1138.186, 1443.784, 2146.147, max

ii - separate quartiles for those 65+
Cut-offs for those below 65 years old: min, 1537.348, 2138.463, 2806.035, max
Cut-offs for those above 65 years old: min, 1102.393, 1342.82, 1832.125, max

iii- separate quartiles for males and females: 1268.992, 1650.207, 2477.892
Cut-offs for males: min, 1268.991, 1650.206, 2477.891, max
Cut-offs for females: min, 1030.964, 1278.591, 1812.999, max

iv - separate quartiles by age cut-off and for males and females
Cut-offs for males below 65 years old: min, 1688.464, 2355.592, 3096.62, max
Cut-offs for males above 65 years old: min, 1239.244, 1524.123, 2164.565, max
Cut-offs for females below 65 years old: min, 1431.664, 1946.587, 2481.063, max
Cut-offs for females above 65 years old: min, 993.5572, 1214.99, 1523.928, max

v - separate quartiles by age groups
Cut-offs for ages 18-29: min, 762.7513, 1330.104, 1863.986, max
Cut-offs for ages 30-34: min, 1415.294, 1916.751, 2526.501, max
Cut-offs for ages 35-39: min, 1547.809, 2075.645, 2625.806, max
Cut-offs for ages 40-44: min, 1732.915, 2280.471, 2921.22, max
Cut-offs for ages 45-49: min, 1711.058, 2262.45, 2924.558, max
Cut-offs for ages 50-54: min, 1631.006, 2180.486, 2857.609, max
Cut-offs for ages 55-59: min, 1577.591, 2158.891 2819.281, max
Cut-offs for ages 60-64: min, 1455.252, 2089.876, 2776.896, max
Cut-offs for ages 65-69: min, 1265.858, 1768.321, 2562.822, max
Cut-offs for ages 70-74: min, 1135.753, 1409.533, 1968.663, max
Cut-offs for ages 75-79: min, 1080.793, 1307.677, 1663.9, max
Cut-offs for ages 80-84: min, 1054.235, 1263.777, 1562.472, max
Cut-offs for ages 85-89: min, 1045.124, 1240.074, 1526.392, max
Cut-offs for ages 90 +: min, 1062.951, 1235.335, 1529.491, max

Indicator: part of household income
I - quantiles based on all individuals (overall)
Cut-offs: min, 1079.73, 1372.048, 1950.124, max

ii - separate quartiles for those 65+
Cut-offs for those below 65 years old: min, 1315.814, 1816.34, 2384.279, max
Cut-offs for those above 65 years old: min, 1058.595, 1289.154, 1768.045, max
